# Insight into the Genome of *Staphylococcus* *xylosus*, a Ubiquitous Species Well Adapted to Meat Products

**DOI:** 10.3390/microorganisms5030052

**Published:** 2017-08-29

**Authors:** Sabine Leroy, Aurore Vermassen, Geoffrey Ras, Régine Talon

**Affiliations:** Université Clermont-Auvergne, INRA, MEDIS, F-63000 Clermont-Ferrand, France; sabine.leroy@inra.fr (S.L.); aurore.vermassen@inra.fr (A.V.); geoffrey.ras@inra.fr (G.R.)

**Keywords:** *Staphylococcus xylosus*, starter, meat, carbohydrate, amino acid, antioxidant properties, osmotolerance, iron homeostasis

## Abstract

*Staphylococcus xylosus* belongs to the vast group of coagulase-negative staphylococci. It is frequently isolated from meat products, either fermented or salted and dried, and is commonly used as starter cultures in sausage manufacturing. Analysis of the *S. xylosus* genome together with expression in situ in a meat model revealed that this bacterium is well adapted to meat substrates, being able to use diverse substrates as sources of carbon and energy and different sources of nitrogen. It is well-equipped with genes involved in osmotic, oxidative/nitrosative, and acidic stress responses. It is responsible for the development of the typical colour of cured meat products via its nitrate reductase activity. It contributes to sensorial properties, mainly by the the catabolism of pyruvate and amino acids resulting in odorous compounds and by the limiting of the oxidation of fatty acids, thereby avoiding rancidity.

## 1. Occurrence of *Staphylococcus xylosus* in Meat Products

*Staphylococcus xylosus* is one of the 41 species belonging to the coagulase-negative group (CNS) of *Staphylococcus* genus affiliated to the phylum of Firmicutes. *S. xylosus* belongs to the *Staphylococcus saprophyticus* cluster group, which includes nine species (www.bacterio.net).

*S. xylosus* is a commensal species of the epithelium and mucous membranes of animals and more particularly of mammals. It was first isolated from human skin, but its occurrence is uncommon in humans compared with small mammals and farm animals [1,2]. The high presence of *S. xylosus* on the skin of livestock could explain its presence in meat.

In fresh sausages, *S. xylosus* accounted for 48% of the isolates followed by *S. equorum* (24%), *S. pasteuri* (13%) and *S. warneri* (9%) [3,4]. A high-throughput approach of 16S DNA pyrosequencing has revealed the presence of CNS including *S. xylosus* in samples of fresh meat, minced steaks of beef or veal, and poultry sausages [5].

Processes such as fermentation, salting and drying select CNS, which reach concentrations of 5 to 7 log CFU/g and constitute, with lactic acid bacteria (LAB), the main populations in this kind of product [6,7]. The ecology of *S. xylosus* in these products has been particularly studied. In traditional Mediterranean fermented sausages, *S. xylosus* often constitutes the dominant staphylococcal species [8,9,10,11,12,13,14,15,16,17]. In addition, species such as *S. equorum*, *S. saprophyticus* and *S. succinus* belong to this staphylococcal ecosystem. *S. xylosus* is also frequently isolated from natural casings used for sausage manufacturing [15,18]. In salted and dried products such as Iberian ham, *S. xylosus* was identified as the main species throughout the manufacturing process [19,20]. Brine was the main source of staphylococci, with *S. xylosus* accounting for 29% of the isolates [21]. Of the nine CNS species identified in Kitoza, a traditional Malagasy product made from dried or smoked pork or beef, *S. xylosus* represented 10 to 17% of the isolates [7].

*S. xylosus* is also one of the main starters inoculated in meat fermented products. In industrial sausage production where *S. xylosus* is used as starter, it remains dominant regardless of the fermentation stage, showing a good adaptation of the bacterium to the meat substrate and to the various manufacturing processes [22,23]. Complete genomes are available for three *S. xylosus* strains: C2a (our laboratory, LN554884), SMQ-121 [24], and HKUOPL8 [25]. Moreover, the draft genomes of two other strains are also available: NJ [26] and DMB3-Bh1 [27]. In this article, we essentially focus on the strain *S. xylosus* C2a.

## 2. Adaptation to Substrates

Meat contains proteins (15–22 g/100 g), lipids (1.5–4.0 g/100 g), minerals and traces of carbohydrates [28]. In sausage manufacturing, carbohydrates are added to favour fermentation. The metabolic potential of the strain *S. xylosus* C2a, the most characterised strain among the species sequenced, is discussed in relation to these substrates.

### 2.1. Carbon Substrates

Sugar transport and catabolism have been well characterised in *S. xylosus* C2a. Two types of transport have been identified: the phosphoenolpyruvate-dependent sugar phosphotransferase system (PTS) and the PTS-independent system [29]. The PTS system consists of two nonspecific energy-coupling components, Enzyme I (EI, *ptsI*) and a heat stable phosphocarrier protein (HPr, *pstH*), and several sugar-specific multiprotein permeases known as enzymes II (EIIA, EIIB and EIIC) [30]. The PTS-independent system consists of a permease and a kinase [31].

Genome-based analyses of the metabolic pathways for energy production reveal that *S. xylosus* possesses the genetic potential for transport of 10 carbohydrates by the PTS system and eight by the non-PTS system (Table 1). We focused on the three main carbohydrates added to the meat batter, glucose, sucrose and lactose, and the lactate already present in meat.

Glucose can be transported by PST-dependent and independent systems in *S. xylosus* (Table 1) [29,30]. In *S. xylosus*, the genes *ptsI* and *ptsH* are co-transcribed forming the *ptsHI* operon (SXYL_01852-53) [32]. In the genome of *S. xylosus* C2a, three genes encoding specific glucose permeases, *ptsG*, SXYL_00253 and *crr*, are present. These genes are not organised in clusters, in contrast to the *glcA* and *glcB* described in *S. carnosus* [33]. In the PTS-independent system, glucose is transported through the membrane by GlcU permease (*glcU*) and then phosphorylated by a glucokinase (*glkA*) [31]. The *gdh* gene encoding a glucose dehydrogenase is downstream of *glcU* [31]. The Gdh enzyme allows the formation of gluconate from glucose and is co-expressed with GlcU. This co-expression suggests that *S. xylosus* uses two metabolic pathways in parallel for energy yield from glucose [31]. The entry of glucose into the cell depends on its concentration in the extracellular medium [29]. GlcU permease is functional at high concentration, whereas the glucose-specific PTS system transport is active at low concentration. The two main routes of catabolism of glucose in staphylococci are the Embden–Meyerhof–Parnas (EMP) pathway and the pentose phosphate (PP) pathway [30]. However, under growth conditions in a complex medium, about 85% of the glucose is catabolised by the EMP route. The main product of the anaerobic metabolism of glucose is lactate, although it is poorly produced in *S. xylosus* [30]. Acetate and CO_2_ are the main products of aerobic glucose metabolism [30,34,35,36]. Staphylococci can adapt to the oxygenation conditions via the EMP and PP pathways, the TCA cycle and their respiratory chain. The availability of carbon substrates leads to a regulatory process called catabolic carbon repression [29,37]. This results in the repression of genes or operons allowing the use of alternative carbon sources. In staphylococci, the catabolic repression of glucose is provided by a catabolite control protein A (CcpA) transcriptional regulator [30].

In a ground pork meat model containing 0.5% glucose and 2.77% of NaCl incubated at 22 °C in Petri dishes, monoculture of *S. xylosus* C2a consumed glucose and lactate simultaneously [38]. The entry of glucose was via the PTS-independent system, and it was catabolised through the EMP and the PP pathways [38]. Lactate was imported by a lactate permease and catabolised to pyruvate by a lactate–quinone oxidoreductase (Table 1). Acetyl-CoA generated by the degradation of glucose and lactate can feed the TCA cycle and, in part, was catabolised to acetate, which was excreted in meat. The genes encoding an ATP synthase (*atp*, SXYL_00824-31) were overexpressed, leading to an energy supply necessary for *S. xylosus* [38]. This simultaneous consumption of glucose and lactate has been observed for *S. aureus* in laboratory media [35].

In *S. xylosus*, four *scr* genes allow the use of sucrose (Table 1) [39]. The *scrA* gene encodes a sucrose PTS permease composed of EIIBC domains. Internalised sucrose-6-phosphate is cleaved by a sucrose phosphate hydrolase or sucrase encoded by the *scrB* gene to yield glucose-6-phosphate and fructose, which will rejoin the glycolysis [39]. The fructose is then phosphorylated by a fructokinase encoded by *scrK* [40]. The genes *scrB* and *scrK* are organised in an operon. The *scrA* gene is independent [40]. The expression of the two genes *scrA* and *scrB* is induced by the concentration of sucrose present in the medium. This regulation is under the control of a repressor encoded by the *scrR* gene, located upstream of the *scrB* gene [41].

In *S. xylosus*, lactose is assimilated, unphosphorylated, by a lactose permease, which transports galactosides and pentoses (Table 1) [42]. The system comprises *lacP* and *lacH* genes, which respectively encode a lactose permease and a β-galactosidase, which hydrolyses lactose to glucose and galactose. A *lacR* regulatory gene is positioned upstream of the operon and oriented opposite to the *lacPH* operon [30,43]. Galactose is further degraded along the Leloir pathway leading to glucose 1-P via the *galKET* cluster with *galR* upstream encoding a regulator.

### 2.2. Nitrogen Substrates, Peptides, Amino Acids

Proteins are the main components of meat. They are hydrolysed mainly into peptides by endogenous proteinases during sausage fermentation and ripening [28,44]. The proteolytic activity of staphylococci is low [45]. The peptides can be further catabolised into amino acids by endogenous and bacterial peptidases [44]. *S. xylosus* used as starter in association with a lactic acid bacterium contributed to the enrichment of free amino acids in sausages [44,46].

We identified, in the *S. xylosus* C2a genome, two clusters of genes encoding oligopeptide transport systems that belong to the ATP-binding cassette (ABC) family of transporters (Table 2). They are composed of five subunits: an extracellular oligopeptide-binding protein, which specifically captures the substrates, two transmembrane proteins forming the pore and two proteins in charge of ATP hydrolysis. At a genetic level, the five genes are always organised in an operon. Bacteria can have two operons that can be transcribed differently [47]. In *S. xylosus*, only the operon SXYL_00298-00302 was upregulated in a meat model [38]. *S. xylosus* C2a has a potentially high genetic capacity to degrade peptides, with 20 genes encoding peptidases (Table 2) and 14 encoding putative peptidases (LN554884). Four of these genes were overexpressed in a meat model and could contribute to the nutrition of *S. xylosus* C2a, *map* and *amps* encoding methionine and leucyl aminopeptidases and the genes encoding U32 family peptidases (SXYL_01247-48) [38].

The genome of *S. xylosus* contains 59 tRNAs for all amino acids. *S. xylosus* is prototrophic, it can grow on a medium containing ammonium sulphate as the sole nitrogen source [48]. In a meat model that contains variable levels of amino acids, 34 genes involved in synthesis of branched chain and aromatic amino acids, histidine and arginine were downregulated and genes involved in transport of alanine and lysine were upregulated [38]. Most of these genes are under the control of CodY, a global repressor of the transcription that controls the genes involved in the utilisation of nitrogen [49], while the genes involved in arginine synthesis are under the control of CcpA [50].

Arginine is commonly found in meat and could be used as an alternative energy source via the arginine deiminase (ADI) pathway. The ADI pathway comprises three enzymatic reactions catalysed by arginine deiminase (ADI) encoded by *arcA*, ornithine transcarbamoylase (OTC) encoded by *arcB* and carbamate kinase encoded by *arcC*. In the three completed genomes of *S. xylosus*, the genes *arcB* and *arcC* are present, but *arcA* encoding the arginine deiminase is absent (Table 2). However, the *arcA* gene has been detected by a PCR approach in 3 out of 13 *S. xylosus* strains, and among these three strains, one had an arginine catabolic mobile element, ACME-associated *arcA* gene [51,52]. Thus, this gene seems to be found infrequently in *S. xylosus* species. Arginine could be also catabolised by arginase encoded by *arg*, a gene frequent in *S. xylosus*, present in the three strains sequenced and in 12 out of 13 strains [51]. Arginase activity leads to ornithine and urea, and urea can be further catabolised by a urease to carbonic acid and two molecules of ammonia serving as a nitrogen source. In a meat model, concentration of arginine decreased after 48 h of incubation and at this time glucose and lactate were exhausted, and genes related to urea pathway were upregulated [53].

Glutamate and glutamine are present in meat. Glutamate is a key component; it serves as an amino group donor and is a link between nitrogen and carbon metabolism. Glutamate can be imported by a glutamate symporter encoded by *gltT* and catabolised by glutamate dehydrogenases (*gluD1*, *gluD2*) that provide alpha-ketoglutarate, which will fuel the TCA cycle [38]. Furthermore, two clusters of genes *gltBCD* and SXYL_00105-108 linked respectively with glutamate and glutamine interconversion were highly overexpressed in meat (Table 2).

### 2.3. Nucleosides

**Figure 1:**
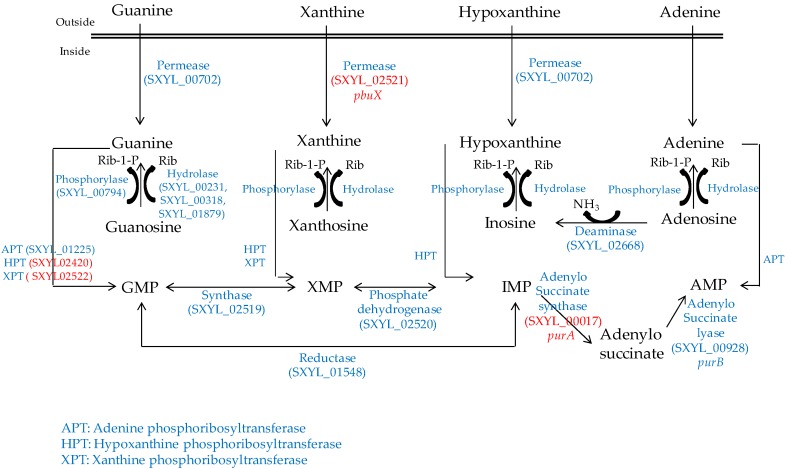
Purine transport, salvage and interconversion in *S. xylosus* C2a. In red, genes overexpressed in a meat model.

**Figure 2:**
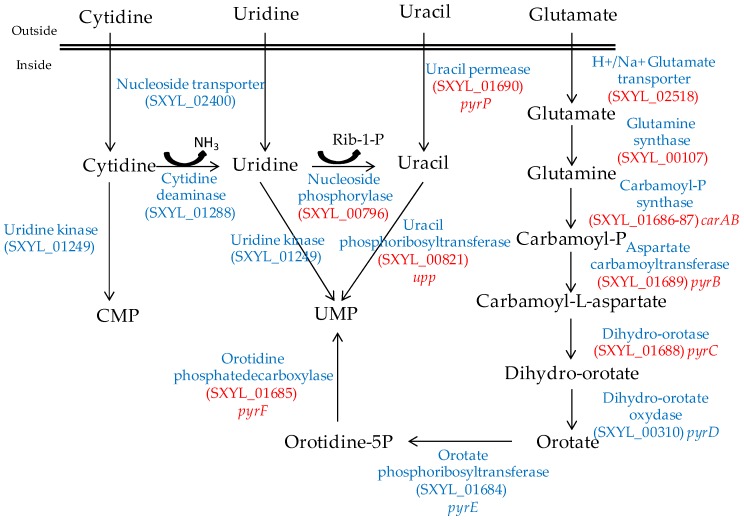
Synthesis of UMP by *S. xylosus* C2a from pyrimidine and glutamate/glutamine. In red, genes overexpressed in a meat model.

Nucleosides are released from the ATP hydrolysis that occurs during the maturation of the meat and during the fermentation process [54]. The *S. xylosus* genome contains the catabolic genes involved in purine and pyrimidine transport, salvage and interconversion (Figure 1 and Figure 2) as described for *Bacillus subtilis* [55]. Purine and pyrimidine bases and nucleosides are transported into the cells by specific permeases and four corresponding genes were identified in *S. xylosus*. The bases are converted to nucleoside monophosphates by phosphoribosyltransferases. Ribonucleosides are cleaved by phosphorylases into free bases and ribose-1 P or by hydrolases into free bases and ribose (Figure 1 and Figure 2). Then, ribose will fuel the PP pathway. The conversion of adenosine to inosine and that of cytidine to uridine are catalysed by specific deaminases releasing NH_3_ that may serve as nitrogen source. The catabolism of inosine and adenosine has been highlighted in coagulase-negative staphylococci [52] and in *Lactobacillus sakei* [56,57].

In meat, intermediates from ATP such as xanthine and uracil are present [58]. *S. xylosus* C2a modulates thirteen genes involved in nucleotide transport and metabolism in a meat model [38]. Xanthine could be imported by the xanthine permease encoded by *pbuX* and catabolised into XMP by the xanthine and hypoxanthine phosphoribosyltransferases encoded by *xpt* and *hpt* (Figure 1). AMP could be synthesised by adenylosuccinate synthase (*purA*) from IMP (Figure 1). Uracil could be taken up via uracil permease *(pyrP*) and catabolised into UMP by uracil phosphoribosyltransferase (*upp*) (Figure 2). Remarkably, UMP could also be synthesised by the import of glutamate or glutamine, which are both present in meat, and then catabolised through six enzymatic reactions encoded by the cluster *pyrEFcarABpyrCB* and *pyrD* (Figure 2).

### 2.4. Iron Uptake

Meat is an iron-rich substrate including hemic (myoglobin and haemoglobin) and non-hemic (ferritin and transferrin) iron sources. *S. xylosus* C2a has developed six systems (*sit*, *sfa*, *hts*, *fhu*, *sst*, SXYL_00561-63) to acquire iron that can be separated into two general mechanisms. The first, represented by *sitABC* (SXYL_02216-18) and the cluster SXYL_00561-63, involves a direct contact between the bacteria and the exogenous sources of iron. The cluster *sitABC*, encoding an iron-regulated ABC transport involved in divalent metal uptake, was influenced in the presence of ferrous iron (FeSO_4_), while the cluster SXYL_00561-63 was highly upregulated in the presence of ferritin [59]. This cluster encodes an oxidoreductase, a monooxygenase and a transporter and was identified in other species belonging to the *S. saprophyticus* cluster group [59]. Remarkably, the Isd (iron responsive surface determinant) system responsible for iron acquisition from heme in *S. aureus* [60] was absent in the three *S. xylosus* sequenced strains; only *isdG* encoding the monooxygenase was present. The second mechanism relies on siderophores, which are small molecules that are secreted by bacteria and have an exceptionally high affinity for iron [61]. It has been clearly demonstrated that *S. aureus* produces two distinct siderophores: staphyloferrin A (SA) and staphyloferrin B [62]. In the *S. xylosus* C2a genome, only the clusters *sfaABCD* and *htsABC* coding for the synthesis and transport of SA were present. The C2a strain was able to produce siderophore in a staphylococcal siderophore detection medium [53]. Furthermore, *S. xylosus* possesses the Fhu system involved in the uptake of hydroxamate-type and the Sst system for catechol-type siderophores. This strain could be able to use exogenous siderophores to scavenge iron from various sources.

## 3. Adaptation to Stressful Manufacturing Processes

### 3.1. Osmotic Stress

*S. xylosus* is consistently isolated from fermented sausages and dry cured meat products. It is able to grow in the presence of curing salts. It is a remarkably osmotolerant bacterium, like other staphylococci. Osmosprotection appears to be crucial in this salted environment. *S. xylosus* has developed several mechanisms to cope with the osmotic stress. It possesses several osmoprotectant systems, such as solute uptakes for proline, serine/alanine/glycine and glycine betaine/carnitine/choline, and synthesis of glycine betaine (Table 3).

The response of *S. xylosus* to the presence of NaCl was studied in the meat model [38]. It responded by under-expressing *mscL* (SXYL_01536), which encodes a mechanosensitive channel, which prevents the efflux of solute. It also involved different mechanisms of accumulation of osmoprotectants and Na^+^-dependent antiporters (Table 3). Thus, *S. xylosus* overexpressed the genes encoding two systems of uptake and synthesis of glycine betaine, a major osmoprotectant. The *opuC* cluster encodes a betaine/carnitine/choline type ABC carrier for the uptake of the carnitine present in meat, which can be catabolised to glycine betaine by an l-carnitine dehydrogenase encoded by *lcdH*. The *cudT*C*A* genes encode enzymes involved in the acquisition of choline and its dehydrogenation to glycine betaine by a choline dehydrogenase encoded by *betA* [63]. In parallel, the two *mnh* clusters encoding Na^+^/H^+^ antiporter systems were overexpressed by *S. xylosus* in the meat model [38].

### 3.2. Oxidative, Nitrosative Stress

Meat processing generates changes in oxygen levels and redox potential. This level varies during mincing, and an oxygen gradient is established during fermentation between the surface and the heart of the sausage. Furthermore, nitrite added during manufacturing undergoes chemical reactions that lead to reactive nitrogen species (RNS) including NO [64]. These RNS and reactive oxygen species (ROS) will generate nitrosative and oxidative stress, especially in meat in which iron contributes to Fenton chemistry generating highly reactive hydroxyl radicals. Staphylococci have several mechanisms to overcome the deleterious effects of this stress [65].

*S. xylosus* C2a possesses 14 genes coding for enzymes involved in the detoxification of ROS and RNS and, in particular, one superoxide dismutase, three catalases, four peroxiredoxins, four nitroreductases and a nitric oxide synthase (Table 4). Nine of them were overexpressed in response to nitrosative stress in a meat model containing curing salts, nitrate and nitrite (Table 4) [53]. *S. xylosus* possesses a single superoxide dismutase (SOD) encoded by *sodA* involved in protection against oxidative stress generated by hyperbaric oxygen and paraquat [66]. The expression of this gene was not modulated in the meat model with or without curing salts [38,53]. In *S. xylosus* C2a, the detoxification of H_2_O_2_ is accomplished by three catalases (KatA, KatB, KatC). The genes encoding these three catalases are also present in the genomes of the strains SMQ-121 and HKUOPL8. Most staphylococci have one catalase, as *S. aureus* [65], but the strain *S. carnosus* TM300 has two [67], as do some strains of *S. equorum*, *S. saprophyticus* and *S. xylosus* [68]. The transcription of *katA* of *S. xylosus* C2a was induced upon entry in the stationary phase, by oxygen and hydrogen peroxide, and was repressed by iron and manganese [69]. This gene was down-regulated, while *katB* and *katC* were upregulated in response to a nitrosative stress in a meat model [53]. In addition to catalases, *ahpC* encoding alkyl hydroperoxide reductase subunit C, *bsaA* encoding glutathione peroxidase and *bcp* encoding a bacterioferritin comigratory protein were upregulated. AhpC confers resistance to ROS and BsaA detoxifies H_2_O_2_ and also other peroxides (ROOR) [65]. Bacterioferritin comigratory protein functions as an iron chelator and is homologous to a thioreductase-peroxidase contributing to the reduction of thiol-dependent peroxides [65]. The genome of *S. xylosus* C2a contains four genes encoding nitroreductases and three were upregulated to cope with nitrosative stress in a meat model with curing salts [53]. These nitroreductases may help to maintain the thiol disulphide balance as shown for *S. aureus* [70].

A gene encoding a nitric oxide synthase (NOS) is present in *S. xylosus* genomes as in all staphylococci. In *S. xylosus* C2a, this enzyme protects against peroxide stress [71]. Furthermore, the loss of NOS activity in this strain resulted in the modulation of the expression of genes encoding catalases, with upregulation of *katA* and downregulation of *katB* and *katC* [71]. In this study, a *nos* deficient mutant displayed higher colony pigmentation than the C2a wild-type strain. All these results are in agreement with those on *S. aureus*, attesting that NOS activity protects against oxidative stress [72,73]. *S. xylosus* C2a grown in a meat model overexpressed the cluster *crtPQMN* (SXYL_00051-54) involved in carotenoid pigment biosynthesis pathway [38]. In *S. aureus*, the pigment protects against oxidative stress by scavenging free radicals [74].

Proteins and amino acids can be oxidised or modified by ROS and RNS. Staphylococci have developed mechanisms to repair protein damage [65]. Thioredoxin and glutaredoxin are essential to maintain protein thiols in their reduced forms. They are major contributors to oxidative stress resistance by facilitating the reduction of H_2_O_2_, scavenging hydroxyl radicals and donating reducing equivalents to peroxiredoxins [65]. The *S. xylosus* C2a genome has four genes encoding thioredoxins and one encoding glutaredoxin (Table 3). The transcription of *trxB* encoding a thioredoxin reductase was increased in the presence of RNS in a meat model [53]. Similarly, stressors such as hydroperoxide and disulphide induce transcription of *trxA* and *trxB* in *S. aureus* [75]. *S. xylosus* C2a, as *S. aureus*, has three *msrA* genes and one *msrB* encoding methionine sulphoxide reductase involved in the repair of oxidised methionine (Table 4). In *S. aureus*, MsrA1 is the major contributor against H_2_O_2_ stress [76]. This gene was also overexpressed in *S. xylosus* to counter nitrosative stress in a meat model [53].

**Figure 3:**
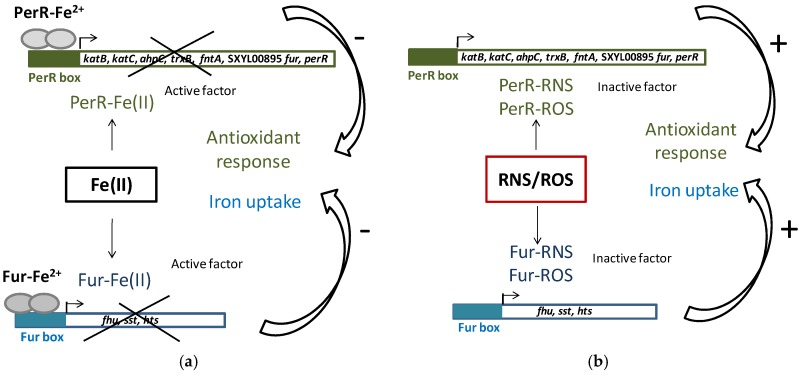
PerR and Fur regulation in *S. xylosus* C2a. (**a**) Classic model of PerR and Fur repression of antioxidant response and iron uptake; (**b**) Impact of reactive nitrogen species (RNS) and reactive oxygen species (ROS), which compete with Fe(II) for PerR and Fur binding, cause antioxidant response and iron uptake.

Iron homeostasis is essential because of its involvement in the generation of ROS. To maintain iron homeostasis, *S. xylosus* C2a, in addition to the six iron-acquiring systems described above, can store iron in ferritin (*ftnA*) and bacterioferritin comigratory protein (*bcp*) (Table 4). The clusters (*fhu*, *sst*, *sfa*, *hts*) and the genes *ftnA* and *bcp* were upregulated in a meat model containing nitrate and nitrite (Table 4) [53]. Some of these genes (*hts*, *fhu*, *sst*) belong to the Fur (ferric uptake regulator) regulon. Fur can be inactivated by NO from nitrite, thus derepressing the regulon (Figure 3) [53]. In addition, *perR* encoding the transcriptional regulator PerR, a member of the Fur family and identified as a peroxide-sensing protein, was upregulated. The genes *katB*, *katC*, *ahpC*, *trxB* and *fntA*, all of which are upregulated in the presence of RNS, are under the control of PerR [53]. PerR, as Fur, can be inactivated by NO from nitrite (Figure 3).

### 3.3. Acid Stress

During sausage fermentation, pH decreases from 5.8 to the range 4.5 to 5.3, depending on the carbohydrate added and the starters inoculated [77]. Bacteria have developed several mechanisms to cope with acidity [78]. One of them relies on proton extrusion by F_1_F_0_-ATPase, which plays a key role in maintaining the internal pH near neutral. A link between ATPase and acid tolerance has been established for several lactic acid bacteria [78]. The cluster *atp* encoding F_1_F_0_-ATPase and *atpI* (SXYL_00823) encoding a putative ATP synthase protein I were highly overexpressed in *S. xylosus* C2a, which has to adapt to the pH 5.9 of a meat model as the inoculum was grown in chemical defined medium at pH 7.0 [38].

*S. xylosus* C2a also highly overexpressed the cluster *dlt* (SXYL_01987-90) involved in d-alanylation of teichoic acids. The degree of d-alanylation varies depending on environmental conditions such as pH, temperature or salt [79]. Inactivation of *dltC* in *Streptococcus mutans* resulted in the generation of an acid-sensitive strain that could not grow below pH 6.5 [80].

In addition, *S. xylosus* C2a can generate ammonia to neutralise acids, in particular via arginase and urease; the cluster *ureDGFECBA* encoding urease was overexpressed in a meat model [53]. Similarly, increased urease activity appeared to be an important factor in the acid defence in *S. aureus* [81]. The gene *vraS* (SXYL_00951) involved in a two-component system was upregulated in *S. xylosus* in a meat model [38]; this gene was also upregulated in *S. aureus* after an acid shock and is involved in the cell wall stimulon response [81]. This production of ammonia could contribute to the pH increase during Mediterranean sausage ripening and to the flavour [6].

## 4. Functional Properties

*S. xylosus* used as starter culture in sausage manufacturing contributes to the development of sensorial quality, in particular the typical cured colour via its nitrate reductase activity, and to flavour, by producing odorous metabolites from pyruvate and amino acid catabolism and limiting oxidation of free fatty acids [6,82,83,84].

### 4.1. Colour Development

The typical cured colour pigment, nitrosomyoglobin, results from a series of reactions involving the formation of nitrogen oxide (NO), which interacts with the iron Fe^2+^ of the cofactor heme of the myoglobin [64]. The substrate to produce NO could be nitrate or nitrite. Nitrite undergoes chemical reactions that lead to NO in the sausage. These reactions are favoured by the acidification caused by lactic acid bacteria. Addition of nitrate leads to its reduction to nitrite by nitrate reductase of staphylococci. *S. xylosus* strains exhibit variable nitrate reductase activity. Sanchez Mainar and Leroy [85] showed in 13 strains of *S. xylosus* that about 1/3 strains have high activity, 1/3 moderate activity and the remaining have little or no nitrate reductase activity. Similarly, among 23 strains of *S. xylosus*, Mauriello and colleagues noted that 57% have a high activity and 17% an intermediate activity [86]. The nitrate reductase of *S. carnosus* is encoded by the *nar* operon [67,87]. This species also has a nitrite reductase, which reduces nitrite to ammonia and is encoded by the *nir* operon [67,88]. These operons are similar in *S. xylosus*. The operon *narGHJI* (SXYL_00539-42) encodes the subunits α, β, δ and γ of the nitrate reductase and the gene *narT* (SXYL_00547) is involved in the transport of nitrate. Upstream, the operon *nir* is composed of five genes *nirR*, *sirA*, *nirB*, *nirD* and *sirB* (SXYL_00531-36) with *nirR* encoding a regulator, *nirBD* encoding the nitrite reductase and *sirA* and *sirB* are necessary for biosynthesis of the siroheme prosthetic group. Downstream from the *nar* operon, the operon *nreABC* (SXYL_00543-45) is involved in the regulation of the operon *nar* and *nir* in anaerobiosis and in the presence of nitrate [89]. Transcription of the operons *nar*, *nir* and *nre* was enhanced in a meat model without added nitrite or nitrate, probably due to anaerobic conditions [38]. When nitrate and nitrite were added to a meat model, reduction of nitrate by *S. xylosus* was mostly achieved after 24 h of incubation [53].

Safety considerations about nitrite and its potential to form carcinogenic nitrosamines have led to the development of alternatives to this additive [64]. The formation of nitrosomyoglobin by *S. xylosus* has been evidenced in laboratory media and meat but the mechanisms remained to be demonstrated [90,91]. This formation could rely on nitric oxide synthase (NOS), which produces NO from arginine. NOS activity was evidenced in *S. xylosus* C2a through nitrosomyoglobin formation [71]. In parallel, this strain forms oxymyoglobin, probably by its capacity to reduce the Fe^3+^ of metmyoglobin to Fe^2+^.

### 4.2. Flavour Development

#### 4.2.1. Pyruvate Catabolism

**Figure 4:**
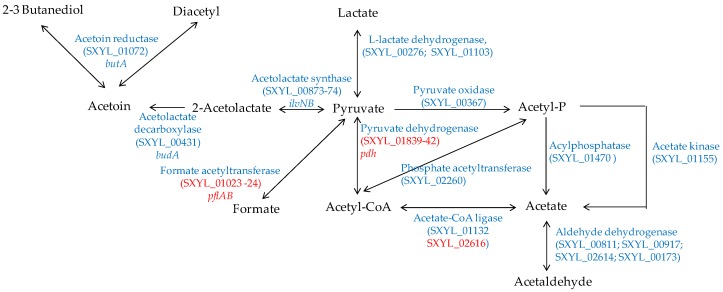
Pyruvate catabolism in *S. xylosus* C2a. In red, genes overexpressed in a meat model.

In a meat model, *S. xylosus* C2a produced mainly acetate from glucose and lactate, which were catabolised simultaneously [38]. Similarly, acetate was found in sausages containing glucose and inoculated by different strains of *S. xylosus* [92,93]. Acetate contributes to the acidic taste and to the aroma in sausage by providing a hint of vinegar [94]. In a meat model, *S. xylosus* C2a catabolised pyruvate to acetyl-CoA by the pyruvate dehydrogenase encoded by the *pdh* cluster, and then to acetate by acetate CoA-ligase (Figure 4) [38]. In addition, *S. xylosus* C2a has the genetic potential to synthesise acetate from acetyl-phosphate originating either from pyruvate or from acetyl-CoA (Figure 4). The formate acetyltransferase encoded by *pflAB* was overexpressed in a meat model, but formate was not measured [38].

*S. xylosus* C2a can synthesise acetoin, diacetyl and butanediol from pyruvate (Figure 4). These compounds with a buttery odour were found in sausages inoculated by *S. xylosus* [92,93]. Acetoin was produced with intraspecies variability by several strains of *S. xylosus* in a meat simulation medium [95]. One of these strains produced acetoin in a northern European sausage and acetoin and diacetyl in a southern European sausage [95].

#### 4.2.2. Amino Acid Catabolism

**Figure 5:**
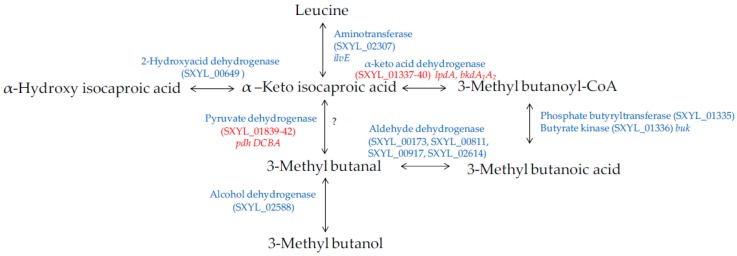
Leucine catabolism in *S. xylosus* C2a. In red, genes differentially expressed in a meat model.

Amino acids, in particular branched-chain amino acids (leucine, isoleucine, and valine), are catabolised into aldehydes, alcohols and acids during sausage manufacturing [94,96]. Methyl-aldehydes with malty odours, methyl-alcohols with fruity odours and methyl-acids with cheesy odours contribute to the aroma of sausages. The catabolism of these amino acids is modulated by *S. xylosus* [92,95,97]. The major metabolite identified was 3-methyl butanol arising from leucine catabolism in minced meat or in sausage [92,95] and in laboratory media [98,99]. The production of this metabolite was substantial during the growth of *S. xylosus* and was observed at different pHs (5.0 to 6.0) and temperatures (20 to 30 °C), parameters relevant for sausage manufacturing [98]. The other metabolites, methyl-aldehyde and methyl-acid, were often identified as minor compounds and depending on the temperature, pH and salt [100,101]. The studies of Beck et al. [102,103] showed that methyl-aldehydes were oxidised into methyl-acids becoming the major metabolites. *S. xylosus* C2a catabolises leucine to alpha-ketoisocaproic acid by a transaminase encoded by *ilvE* (Figure 5). Then, a cluster of 6 genes (SXYL_01335-40) could be involved in the formation of 3-methyl butanoic acid. First, the branched-chain alpha-keto acid dehydrogenase complex leads to the formation of 3-methyl butanoyl-CoA, and phosphate butyryltransferase and butyrate kinase lead to 3-methylbutanoic acid. In *S. xylosus* C2a, the cluster SXYL_01337-40 was overexpressed in a meat model [38]. From 3-methylbutanoic acid, 3-methylbutanal can be formed by aldehyde dehydrogenase and then 3-methylbutanol by alcohol dehydrogenase (Figure 5). We did not find any gene encoding a branched-chain keto acid decarboxylase, thus it seemed that the formation of 3-methyl butanal from alpha-ketoisocaproic acid is not possible by *S. xylosus* C2a, and this does not confirm the hypothesis of Beck et al. [102]. Eventually, the pyruvate dehydrogenase could catalyse the decarboxylation of alpha-ketoisocaproic as described for *Bacillus subtilis* [104].

#### 4.2.3. Lipolysis and Fatty Acid Oxidation

Lipolysis occurs during sausage manufacturing and releases mostly long-chain fatty acids and is to a great extent due to endogenous triglyceride lipases and phospholipases [6,105]. *S. xylosus* can contribute to lipolysis as most strains were able to hydrolyse pork fat [86]. For *S. xylosus*, two lipases have been described: SXL and GehM, which share 53% identity [106,107]. Extracellular lipase SXL alone is well characterised. It is a monomeric protein (43 kDa), which is almost identical to the lipases of *Staphylococcus aureus* and *Staphylococcus simulans.* Its peak activity is at pH 8.2 and 45 °C, it is able to hydrolyse triacylglycerols without chain length specificity, and it is stable between pH 5 and 8.5 and thermostable [107]. A second lipase SXL named SXL2 shares 98.7% identity with SXL [108]. It is also an alkaline lipase (pH 8.5) which acts at high temperature (55 °C) and hydrolyses preferentially short-chain substrates. The structural stability of SXL lipase is modulated by Zn^2+^ ions [109]. The SXL genes are not present in the three completed genomes of *S. xylosus* strains. The lipase GehM is thermostable with a peak activity at pH 9 and 42 °C [110]. The expression of *gehM* was downregulated by the presence of triglycerides in the culture medium [111]. *gehM* is present in the genome of SMQ-121 and HKUOPL8 strains, but is a pseudogene in the strain C2a. In the genome of the strain *S. xylosus* C2a, five genes encoding putative lipases, one encoding a putative phospholipase and three encoding putative lysophospholipases are present (LN554884).

The oxidation of unsaturated fatty acids released by lipolysis results in numerous volatile compounds, including aldehydes, alcohols and ketones, which contribute to the aroma of the sausages [6,96]. This oxidation is essentially a chemical peroxidation via ROS. As underlined above, *S. xylosus* is well equipped to detoxify ROS and thus to limit this fatty acid oxidation. Indeed, it was able to inhibit the oxidation of linoleic and linolenic unsaturated fatty acids in laboratory media [112]. Furthermore, mutants deficient in SOD or catalase activity of *S. xylosus* C2a were less efficient than the wild type in limiting oxidation of unsaturated fatty acids [113].

## 5. Conclusions

The genome analysis of *S. xylosus* used as a meat starter culture together with transcriptomic approaches in situ in meat have highlighted that this bacterium has all functions necessary for its adaptation to meat substrates and to technological stress, and has the potential to contribute to the sensorial quality of sausages. Nevertheless, these functional properties vary according to the strains. Furthermore, the selection of strains for starter cultures should include safety criteria, such as the lack of production of biogenic amines [82,83,114] and enterotoxins and of transferable antibiotic resistance genes [82,114].

## Figures and Tables

**Table 1:** Carbohydrate transport and catabolism in *S. xylosus* C2a.

Phosphotransferase System (PTS)			PTS-Independent		
**Glucose**			**Glucose**		
SXYL_00369	*ptsG*	EIICBA	SXYL_00698	gdh	Glucose 1-dehydrogenase
SXYL_00253		EIIBC	SXYL_00699	glcU	Glucose uptake protein
SXYL_01421	*crr*	EIIA	SXYL_01308	glkA	Glucokinase
**Sucrose**			**Lactate**		
SXYL_00555	*scrA*	EIIBC	SXYL_00250		Lactate permease
SXYL_00886	*scrR*	Repressor	SXYL_00577		Lactate permease
SXYL_00887-88	*scrBK*	Hydrolase, Fructokinase	SXYL_00170		lactate-quinone oxidoreductase
**others**			**Lactose**		
SXYL_00060, SXYL_00626		Arbutin, EIIBC	SXYL_00082	*lacR*	Transcription activator
SXYL_00528		Beta-glucoside, EIIABC	SXYL_00083-84	*lacPH*	Permease, Beta-galactosidase
SXYL_00257-60		Cellobiose, EIIC, EIIBA	SXYL_00671	*galR*	Transcriptional regulator
SXYL_02148-50	*fruAK*	Fructose, EIIABC, catabolism, repression	SXYL_00672-74	*galKET*	Galactokinase, epimerase, P-uridylyltransferase
SXYL_00277-78		Fructose, regulation, EII			
SXYL_00773-76	*mtlD*, *A*	Mannitol, EIIACB, catabolism, regulation	**others**		
SXYL_02255		Maltose, EIICB	SXYL_00122-26	*araRBDAT*	Arabinose, transport, catabolism, regulation
SXYL_01138		*N*-acetylglucosamine, EIIBC	SXYL_01576-78, SXYL_01581	*glpDKF*, *P*	Glycerol, transport, catabolism, regulation
SXYL_02455-57		Trehalose, regulation, catabolism, EIIBC	SXYL_00438-40	*gntRKP*	Gluconate, transport, catabolism, regulation
			SXYL_00159, SXYL_02351		Gluconate, transport
			SXYL_01518-22	*rbsBCADR*	Ribose, transport, catabolism, regulation
			SXYL_00132-35	*xylEBAR*	Xylulose, transport, catabolism, regulation

In red, genes overexpressed in a meat model.

**Table 2:** Peptide transport, peptidases and amino acid catabolism in *S. xylosus* C2a.

Peptide transport	SXYL_00298-302, SXYL_01936-40 (*oppAFDCB*)
Peptidases	SXYL_00314 (*sspA*), SXYL_00502 *(pcp*), SXYL_00620, SXYL_00948 (*map*), SXYL_00957 (*ampS*), SXYL_01078, SXYL_01082, SXYL_01120 (*pepA*), SXYL_01136, SXYL_01247-48, SXYL_01324, SXYL_01348, SXYL_01489, SXYL_01511, SXYL_01806, SXYL_01931, SXYL_01980 (*ampA*), SXYL_02073, SXYL_02105 (*pepT*)
Arginine catabolism	SXYL_00252 (*arcB*), SXYL_02488 (*arcC*), SXYL_00769 *(arg*), SXYL_00290-97 (*ureDGFECBA*)
Glutamate catabolism	SXYL_02518 (*gltT*), SXYL_01964 (*gluD1*), SXYL_02326 (*gluD2*), SXYL_02459-61 (*gltBCD*), SXYL_00105-108

In red, genes overexpressed in a meat model.

**Table 3:** Potential of *S. xylosus* C2a to cope with osmotic stress.

Proline Uptake	SXYL_00427 (*putP1*), SXYL_00935 (*putP2*)
Serine/alanine/glycine uptake	SXYL_01171 (aapA), SXYL_00317
Glycine betaine/carnitine/choline uptake and glycine betaine synthesis	SXYL_00488-91 (*opuCABCD*), SXYL_00486 (*lcdH*), SXYL_00223-26 (*cudTCA*, *betA)*, SXYL_00743 (*opuD2*), SXYL_01535 (*opuD1*), SXYL_01095, SXYL_02127-28
Na^+^/H^+^ antiporter	SXYL_01970-76 (*mnhA1B1C1D1E1F1G1*), SXYL_02220-26 (*mnhG2 F2E2D2C2B2A2*)

In red, genes overexpressed in a meat model.

**Table 4:** Potential of *S. xylosus* C2a to cope with oxidative/nitrosative stress.

Detoxifying Enzymes		Protein Damage Repair		Iron Homeostasis	
SXYL_01303 (*sodA*)	Superoxide dismutase [Mn/Fe]	SXYL_00374	Thioredoxin	SXYL_00747-50 (*sfaDCBA*)	Siderophore biosynthesis Staphyloferrin A
SXYL_02505 (*katA*)	Catalase A	SXYL_01797 (*trxA*)	Thioredoxin	SXYL_00751-3 (*htsABC*)	Iron compound ABC transporter, Staphyloferrin A *
SXYL_01551 (*katB*)	Catalase B *	SXYL_02083 (*trxB*)	Thioredoxin reductase *	SXYL_02113-16 *(sstDCBA*)	Iron compound ABC transporter, Staphyloferrin B *
SXYL_02533 *(katC*)	Catalase C *	SXYL_00519	Thioredoxin-like protein	SXYL_02201-3 *(fhuGBC*)	ABC-type cobalamin/Fe3^+^-siderophores transport system *
SXYL_01572 (*bsaA*)	Glutathione peroxidase	SXYL_01851	Glutaredoxin	SXYL_02681 *(fhuD2*)	Iron(3^+^)-hydroxamate-binding protein *
SXYL_01153 (*tpx*)	Thiol peroxidase	SXYL_01517 (*msrA1*)	Peptide methionine sulphoxide reductase	SXYL_00667 *(fhuD1*)	Iron(3^+^)-hydroxamate-binding protein *
SXYL_02534-35 (*ahpCF*)	Alkyl hydroperoxide reductase *	SXYL_01516	Regulator MsrR	SXYL_00944 *(ftnA*)	Ferritin *
SXYL_00973 (*bcp*)	Bacterioferritin comigratory protein Thioreductase peroxidase	SXYL_0019-21 (*msrA2BA3*)	Peptide methionine sulphoxide reductase	SXYL_00973 (*bcp*)	Bacterioferritin comigratory protein
SXYL_02021	Nitroreductase				
SXYL_00229	Nitroreductase				
SXYL_00410	Nitroreductase				
SXYL_00895	Nitroreductase *				
SXYL_00923 *(nos*)	Nitric oxide synthase				

* Genes under PerR or Fur regulation. In red, genes overexpressed in a meat model with curing salts.
